# Lateral entry pins and Slongo’s external fixation: which method is more ideal for older children with supracondylar humeral fractures?

**DOI:** 10.1186/s13018-021-02541-z

**Published:** 2021-06-21

**Authors:** Man He, Qian Wang, Jingxin Zhao, Yu Jin, Yu Wang

**Affiliations:** 1 Department of Rehabilitation, Affiliated Hospital of Chengde Medical College, Chengde, Hebei 067000 People’s Republic of China; 2 Department of Orthopedics, Affiliated Hospital of Chengde Medical College, 36 Nanyingzi Street, Shuangqiao District, Chengde, Hebei 067000 People’s Republic of China

**Keywords:** Supracondylar humeral fracture, Older children, Kirschner wire, External fixation

## Abstract

**Objective:**

The standard surgical treatment for supracondylar humeral fractures in children is closed reduction and percutaneous pinning. Given the need for greater fixation strength and higher risk of joint stiffness for children older than 8 years, external fixation is often performed for treating supracondylar humeral fractures in older children. The aim of this study was to compare the efficacy of lateral entry pins and Slongo’s external fixation for treating supracondylar humeral fractures in older children.

**Methods:**

Children older than 8 years who underwent surgery for supracondylar humeral fractures at our hospital for surgery from January 2016 to December 2020 are to be retrospectively assessed. One group (n = 36) underwent internal fixation and percutaneous pinning with three lateral Kirschner wires, and the other group (n = 32) underwent Slongo’s external fixator surgery. The demographic data, operation duration, number of fluoroscopies, and fracture healing time were compared between both groups. The elbow joint function was evaluated 6 months after the surgery on the basis of fracture healing time, lifting angle, elbow joint range of motion (ROM), and Flynn score. The incidence of postoperative complications was also recorded.

**Results:**

There was no significant difference between the two patient groups in terms of the demographic parameters. Compared to external fixation surgery, Kirschner wire surgery required shorter duration and fewer fluoroscopies (P < 0.05). Nevertheless, the fracture healing time was significantly less (P < 0.05), and the elbow ROM and Flynn scores were higher in the external fixator group compared to the Kirschner wire fixation group (P < 0.05). There was one case of secondary fracture displacement in the Kirschner wire group and one of pin tract infection in the external fixator group. No other iatrogenic injuries or complications were observed.

**Conclusion:**

Maybe Slongo’s external fixator is a suitable alternative treatment option for supracondylar humeral fractures in children older than 8 years since it can achieve better fixation strength and early restoration of elbow joint movement with a lower risk of joint stiffness.

**Supplementary Information:**

The online version contains supplementary material available at 10.1186/s13018-021-02541-z.

## Background

Supracondylar humeral fractures account for 50–60% of all elbow fractures and 30% of all upper limb fractures in children [1, 2]. They are classified into the extension and flexion types, of which the former is more prevalent (97–99%) [3]. In addition, based on the degree and direction of displacement, the supracondylar humeral fractures are also classified into three types according to the modified Gartland system. Surgical treatment is usually recommended for Gartland II and III fractures in children [4]. The standard surgical treatment for displaced pediatric supracondylar humeral fractures [5, 6] is closed reduction and percutaneous pinning.

Age is a key factor determining the occurrence and features of supracondylar humeral fractures and also affects the functional recovery of the elbow joint [7, 8]. Older children are defined as those > 1 standard deviation from the mean (8 years) [9]. While these fractures are most common in children aged 5–7 years, the degree of fracture displacement is more serious in those older than 8 years, resulting in a higher risk of neurovascular injury, open fractures [1, 10], and joint stiffness. Therefore, recovery of the elbow joint is slower in older children [1, 11], and rehabilitation is often needed to restore normal joint function [7].

Supracondylar humeral fractures in older children require firmer fixation since percutaneous pinning increases the chances of joint stiffness [9, 12]. The external fixator technique was modified by Slongo et al. to treat pediatric supracondylar humeral fractures and has the advantages of good fixation strength, lower risk of compartment syndrome due to swelling, and early functional exercise. In this study, we retrospectively compared the efficacy of lateral entry pins and Slongo’s external fixation in the treatment of supracondylar humeral fractures in older children.

## Methods

### Patients

The clinical data of 68 children older than 8 years with supracondylar humeral fractures who were treated at the Affiliated Hospital of Chengde Medical College from January 2016 to December 2020 was retrospectively analyzed. Inclusion criteria for this study were aged between 8 and 14 years, Gartland types II and III, and flexion-type supracondylar humeral fractures. Children with open fractures or fractures in other bones or organ injuries were excluded. Thirty-six children were treated with three Kirschner wires inserted laterally, and 32 children were treated with Slongo’s external fixator. The operation duration and number of fluoroscopies were recorded. The demographic and clinical characteristics of the patients are summarized in Table 1. Informed consent was obtained by the parents or guardians. The study was reviewed and approved by the institutional ethics committee.

**Table 1:** Basic condition of patients

Group	Kirschner wire	External fixation	***P***
Gender (male/female)*	25:11	20:12	0.546
Age (years old)	10.58 ± 1.63	11.14 ± 1.55	0.792
Height (cm)	121.33 ± 21.99	108.39 ± 23.26	0.860
Weight (kg)	32.95 ± 16.96	35.63 ± 12.68	0.140
Affected side (example, left/right)	20:16	15:17	0.475
Type (example)*			0.284
Gartland II	14 (38.9%)	6 (18.8%)	
Gartland III	21 (58.3%)	24 (75%)	
Flexion type	1 (2.8%)	2 (6.3%)	
Time from admission to operation (h)	35.81 ± 27.96	59.69 ± 38.97	0.151
Hospitalization time (d)	3.81 ± 0.17	4.78 ± 0.26	0.807
Operation time (min)	25.09 ± 4.15	50.86 ± 3.38	0.037
Number of intraoperative fluoroscopy (time)	16.81 ± 2.83	22.97 ± 1.86	0.022
Time for removal of internal fixation (weeks)	5.04 ± 0.37	4.07 ± 0.22	0.006

*Use the chi-square test to calculate the *P* value

### Surgical technique

**Fig. 1:**
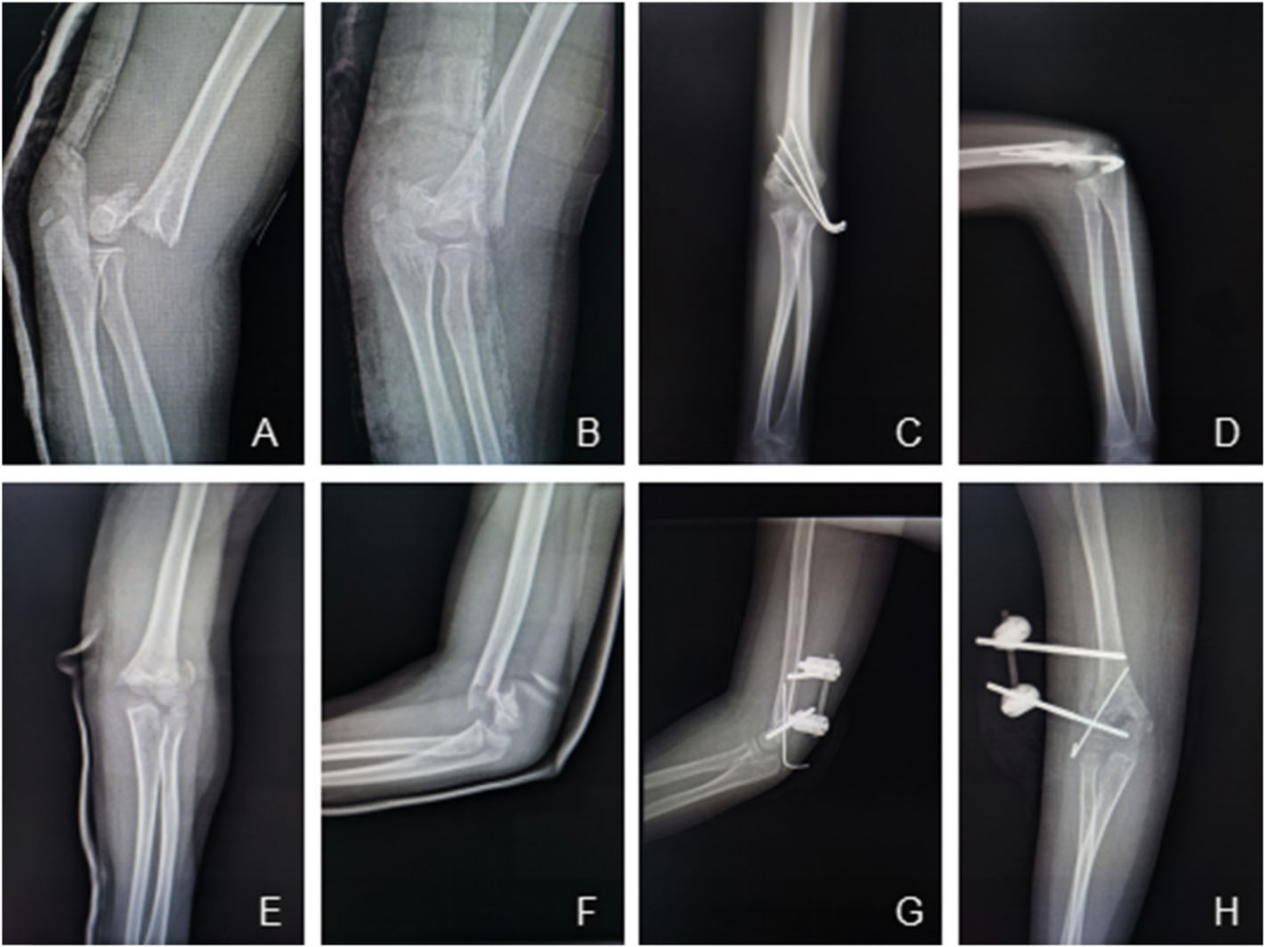
**A**, **B**, **E**, **F** Two cases of extension-type supracondylar fracture of the humerus in older children. **C**, **D** X-ray of the elbow joint after internal fixation with Kirschner wire. **G**, **H** X-ray of elbow joint after external fixation

The operation was performed under general anesthesia. The patient was placed in a supine position, and the affected limb was placed on a C-arm fluoroscopy machine. The fracture reduction was performed under C-arm fluoroscopy. Briefly, a 2.5-mm Kirschner wire was placed parallel to the epiphyseal plate of the lateral humerus condyle based on the elbow joint X-ray to avoid screw damage to the epiphysis and epiphyseal plate of the external condyle. The proximal fracture end was opened with a small incision to expose the humeral bone, and a 4-mm cancellous self-drilling Schanz screw was carefully inserted into the humerus while avoiding the coronal fossa, olecranon fossa, and medial bone cortex of the distal humerus. A sleeve was used to protect the surrounding soft tissue and ensure that the screw was located in the center of the humerus. Manual closed reduction was performed with the Schanz screw acting as a joystick. After satisfactory reduction was achieved, the connecting rod was used to fix the screw. Finally, a 1.8-mm or 2-mm Kirschner wire was inserted retrograde percutaneously into the lateral condyle of the humerus to prevent rotation of the fractured end. The firmness of the fixation and elbow movement were checked (Fig. 1).

### Postoperative treatment, reexamination, and evaluation

Clinical and imaging tests were conducted 2 weeks, 4 weeks, 2 months, 3 months, and 6 months after the operation. The Kirschner wire, plaster, or external fixator were removed once the fracture has healed, and the healing time of the two groups was recorded. The patients with preoperative neurovascular injury were followed up until the symptoms disappeared. Elbow function was evaluated after 6 months by measuring the ROM and carrying angle of the elbow joint, and the Flynn scores were calculated for both groups. In addition, postoperative complications such as infection, iatrogenic nerve injury, myositis ossificans and deformity, vascular sensation, and osteofascial compartment syndrome were also recorded.

### Statistical analysis

The data were analyzed by SPSS 22.0 software. Descriptive statistics including means and frequencies were calculated for each of the examined variables. The treatment outcomes of the two surgical methods were compared using an independent-sample t-test for metrological data or chi-square test for the counting data as appropriate. *P* < 0.05 was considered statistically significant.

## Results

Since January 2016, 408 supracondylar humeral fractures have been treated at our hospital, including 123 cases (30%) for children older than 8 years. As shown in Table 1, there was no significant difference between the two surgery groups in terms of basic clinical and demographic characteristics. Compared to external fixation surgery, percutaneous pinning required less time and fewer fluoroscopies (P < 0.05). The fracture healing time was significantly shorter in the external fixator group compared to the Kirschner wire group (P < 0.05), and the elbow ROM and Flynn scores were significantly higher in the former (P < 0.05; Tables 2 and 3). There was one case of secondary fracture displacement in the Kirschner wire group, and one case of superficial pin tract infection around the Schanz screw in the external fixator group. The infection subsided 1 week after the local dressing change. No nail path infection occurred at the anti-rotation Kirschner wire site, and neither severe infection nor osteomyelitis was observed. Any nerve injury recovered spontaneously without nerve exploration. One child in the Kirschner wire group had mild elbow valgus deformity but did not need any special treatment. It might be caused by the secondary displacement of fracture due to the large weight of the child and insufficient Kirschner wire fixation strength. Iatrogenic nerve injury and compartment syndrome were not observed.

**Table 2:** Comparison of elbow joint function in children 6 months after operation

Index (nd	Kirschner wire		External fixation		Affected side ***P***
	Affected side	Contralateral side	Affected side	Contralateral side	
Flexion ROM	140.76 ± 4.38	146.25 ± 3.64	141.57 ± 3.15	146.23 ± 4.25	0.042
Extension ROM	2.30 ± 0.68	−2.66 ± 2.45	2.85 ± 0.48	−2.56 ± 1.01	0.047
Total ROM	141.75 ± 6.03	145.52 ± 5.47	142.34 ± 3.27	145.62 ± 5.25	0.032
Carrying angle (CA)	8.89 ± 0.82	11.13 ± 0.82	8.52 ± 1.07	10.94 ± 0.83	0.300

**Table 3:** Comparison of Flynn scores of the elbow joint in children 6 months after operation

Flynn score	Kirschner wire	External fixation	***p***
According to CA*			0.032
Excellent	5 (15.6%)	15 (41.7%)	
Good	26 (81.3%)	20 (55.6%)	
Fair	1 (3.1%)	1 (2.8%)	
Poor	0	0	
According to the total range of elbow motion loss*			0.033
Excellent	3 (9.4%)	12 (33.3%)	
Good	28 (87.5%)	23 (63.9%)	
Fair	1 (3.1%)	1 (2.8%)	
Poor	0	0	

*Use the chi-square test to calculate the *P* value

## Discussion

Although closed reduction and percutaneous pinning is the standard treatment for supracondylar humeral fractures, external fixation is a more appropriate surgical approach in older children to achieve good reduction and stable fixation, heal the fracture smoothly, and reduce iatrogenic injuries and complications.

There is no current consensus regarding the best pin configuration [13–15]. While one study reported greater mechanical stability of crossed Kirschner wires [16], multiple clinical studies have not observed any significant difference between crossed wire and lateral wire on the outcome of supracondylar humeral fractures in children [13, 17–19]. If two lateral Kirschner wires are used, the first Kirschner wire is placed parallel to the lateral cortex of the humeral shaft metaphysis, and the second Kirschner wire is separated from the first one and fixed on the medial edge of the coronary fossa. The wire insertion point is located on the non-articular surface of the humeral head to avoid hindering elbow joint movement and provide good fixation strength. To ensure the strength of the lateral wire fixation, the lateral column and the middle column of the distal humerus need to be fixed [19]. A third Kirschner wire is often used to further increase the fixation strength [20]. The Kirschner wires should be as dispersed as possible in the sagittal and coronal positions [12]. The lateral wire minimizes the risk of iatrogenic ulnar nerve injury [5, 19], which can be further reduced by avoiding wire insertion from the inside [17]. Thus, lateral pinning is the preferred approach for supracondylar humeral fractures in children [5, 21]. Gartland type II fractures are usually fixed with two lateral Kirschner wires, and type III fractures with three lateral Kirschner wires [22]. In addition, three lateral Kirschner wires are also recommended for children older than 8 years [19, 23, 24].

Kirschner wire fixation may be more suitable for younger children since its limited strength and the need for postoperative plaster fixation increase the risk of secondary displacement and joint stiffness in older children. The external fixator was first used to treat pediatric supracondylar humeral fractures by Taller [25] in 1986. There are three types of external fixation frameworks that are currently used to treat supracondylar humeral fractures in children. Gris et al. [26] and Bogdan et al. [27] used the cross-joint approach to fix the humerus and ulna and treat supracondylar humeral fractures in children. Cross-joint fixation allows early pronation and supination of the forearm, but not early elbow flexion and extension. Slongo [28] placed a Schanz screw at both ends of the fracture and inserted an anti-rotation Kirschner wire laterally. Finally, Gugenheim [29] used the Ilizarov ring external fixator to treat supracondylar humeral fractures in children and adolescents and found its application relatively cumbersome. Slongo’s external fixator has greater stability compared to the crossed Kirschner wire [30], and the operation is relatively simple. In addition, the Schanz screw can be used as a joystick to assist fracture reduction during the operation. Although Slongo’s method required longer operation and more fluoroscopies compared to Kirschner wire fixation alone, it led to faster recovery of joint function and mobility.

Iatrogenic radial nerve injury should be avoided when inserting the Schanz screw at the proximal end of the fracture. Horst et al. [31] suggested that the proximal Schanz screw should be inserted under direct vision within 2 cm from the fracture line and a protective sleeve should be used. We used a 4-mm distal Schanz screw, placed it under fluoroscopy, and inserted a 2.5-mm Kirschner wire into the distal humerus to ensure that the screw did not penetrate the joint and prevent iatrogenic epiphyseal plate and epiphyseal injury. After drilling the screw, a second 1.8-mm or 2-mm Kirschner wire was inserted retrograde into the lateral condyle of the humerus to prevent rotation of the fracture end. Since Schanz screws can obstruct the insertion of the anti-rotating Kirschner wire, the latter must be located under fluoroscopy. The optimal location of the Kirschner wire is behind the distal screw and below the proximal screw, ensuring no tension in the skin around the end of the Schanz screw. In this study, pin tract infection occurred in one case of the external fixation group, which may be related to the local skin tension and the larger diameter of the Schanz screw. Although some studies have shown that the mechanical effect of inserting anti-rotating Kirschner wire from the inside is better [32], there is still a risk of iatrogenic ulnar nerve injury [33]. Therefore, we recommended the lateral entry anti-rotating Kirschner wire.

The limitations of this study are the single-center retrospective design and small sample size. Our findings will have to be validated on a larger cohort with longer follow-up.

## Conclusion

Maybe Slongo’s external fixator is a suitable alternative treatment option for supracondylar humeral fractures in children older than 8 years since it can achieve better fixation strength and early restoration of elbow joint movement with a lower risk of joint stiffness.

## Supplementary Information

**Additional file 1.** During operation.

**Additional file 2.** During operation.

**Additional file 3.** During operation.

**Additional file 4.** During operation.

**Additional file 5.** During operation.

**Additional file 6.** During operation.

**Additional file 7.** Post-operation.

## Data Availability

All data generated or analyzed during this study are included in this manuscript.

## Abbreviations

ROM: Range of motion

## References

[1] Holt JB, Glass NA, Shah AS (2018). Understanding the epidemiology of pediatric supracondylar humeral fractures in the United States: identifying opportunities for intervention. J Pediatr Orthop.

[2] Memisoglu K, Kesemenli CC, Atmaca H (2011). Does the technique of lateral cross-wiring (Dorgan’s technique) reduce iatrogenic ulnar nerve injury?. In Orthop.

[3] Duffy S, Flannery O, Gelfer Y, et al. Overview of the contemporary management of supracondylar humeral fractures in children. Eur J Orthop Surg Traumatol. 2021. 10.1007/s00590-021-02932-2. 10.1007/s00590-021-02932-2 PMC8233294 33744996

[4] Mulpuri K, Hosalkar H, Howard A (2012). AAOS clinical practice guideline: the treatment of pediatric supracondylar humerus fractures. J Am Acad Orthop Surg.

[5] Sinikumpu JJ, Pokka T, Sirviö M (2017). Gartland type II supracondylar humerus fractures, their operative treatment and lateral pinning are increasing: a population-based epidemiologic study of extension-type supracondylar humerus fractures in children. Eur J Pediatr Surg.

[6] Helenius I, Lamberg TS, Kääriäinen S (2009). Operative treatment of fractures in children is increasing: a population-based study from Finland. J Bone Joint Surg Am Volume.

[7] Li M, Xu J, Hu T, Zhang M, Li F (2019). Surgical management of Gartland type III supracondylar humerus fractures in older children: a retrospective study. J Pediatr Orthopaedics B.

[8] Lee S, Park MS, Chung CY (2012). Consensus and different perspectives on treatment of supracondylar fractures of the humerus in children. Clin Orthop Surg.

[9] Fletcher ND, Schiller JR, Garg S, Weller A, Larson AN, Kwon M, Browne R, Copley L, Ho C (2012). Increased severity of type III supracondylar humerus fractures in the preteen population. J Pediatr Orthop.

[10] Holt JB, Glass NA, Shah AS (2018). Understanding the epidemiology of pediatric supracondylar humeral fractures in the United States: identifying opportunities for intervention. J Pediatr Orthop.

[11] Spencer HT, Wong M, Fong YJ, Penman A, Silva M (2010). Prospective longitudinal evaluation of elbow motion following pediatric supracondylar humeral fractures. J Bone Joint Surg.

[12] Segal D, Cobb L, Little KJ (2020). Fracture obliquity is a predictor for loss of reduction in supracondylar humeral fractures in older children. J Pediatr Orthop B.

[13] Picado AV, Morán GG, Moraleda L (2018). Management of supracondylar fractures of the humerus in children. EFORT Open Rev.

[14] Novais EN, Andrade MAP, Gomes DC (2013). The use of a joystick technique facilitates closed reduction and percutaneous fixation of multidirectionally unstable supracondylar humeral fractures in children. J Pediatr Orthop.

[15] St. Clair JB, Schreiber VM (2019). Supracondylar humerus fractures. Oper Tech Orthop.

[16] Eberhardt O, Fernandez F, Ilchmann T, Parsch K (2007). Cross pinning of supracondylar fractures from a lateral approach. Stabilization achieved with safety. J Child Orthop.

[17] Maity A, Saha D, Roy D (2012). A prospective randomised, controlled clinical trial comparing medial and lateral entry pinning with lateral entry pinning for percutaneous fixation of displaced extension type supracondylar fractures of the humerus in children. J Orthop Surg Res.

[18] Yen YM, Kocher MS (2008). Lateral entry compared with medial and lateral entry pin fixation for completely displaced supracondylar humeral fractures in children. J Bone Joint Surg Am.

[19] Kocher MS, Kasser JR, Waters PM, Bae D, Snyder BD, Hresko MT, Hedequist D, Karlin L, Kim YJ, Murray MM, Millis MB, Emans JB, Dichtel L, Matheney T, Lee BM (2007). Lateral entry compared with medial and lateral entry pin fixation for completely displaced supracondylar humeral fractures in children. J Bone Joint Surg.

[20] Hamdi A, Poitras P, Louati H, Dagenais S, Masquijo JJ, Kontio K (2010). Biomechanical analysis of lateral pin placements for pediatric supracondylar humerus fractures. Jo Pediatr Orthop.

[21] Skaggs DL, Hale JM, Bassett J (2001). Operative treatment of supracondylar fractures of the humerus in children: the consequences of pin placement. J Bone Joint Surg.

[22] Vuillermin C, May C, Kasser J (2018). Closed reduction and percutaneous pinning of pediatric supracondylar humeral fractures. Jbjs Essential Surg Tech.

[23] Jacob Weinberg Mohan VB (2010). The role of lateral-entry Steinmann pins in the treatment of pediatric supracondylar humerus fractures. J Childrens Orthop.

[24] Guy SP, Ponnuru RR, Gella S (2011). Lateral entry fixation using three divergent pins for displaced paediatric supracondylar humeral fractures. Isrn Orthop.

[25] Taller S (1986). Use of external fixators in the treatment of supracondylar fractures of the humerus in children. Acta Chir Orthop Traumatol Cech.

[26] Gris M, Nieuwenhove OV, Gehanne C (2004). Treatment of supracondylar humeral fractures in children using external fixation. Orthopedics.

[27] Bogdan A, Quintin J, Schuind F (2016). Treatment of displaced supracondylar humeral fractures in children by humero-ulnar external fixation. Int Orthop.

[28] Slongo T (2008). Lateral external fixation--a new surgical technique for displaced unreducible supracondylar humeral fractures in children. J Bone Joint Surg Am.

[29] Gugenheim Jr JJ.2000, The Ilizarov fixator for pediatric and adolescent supracondylar fracture variants.J Pediatr Orthoped. 20(2):177. 10739278

[30] Hohloch L, Konstantinidis L, Wagner FC, Strohm PC, Südkamp and NP, Reising K (2015). Biomechanical evaluation of a new technique for external fixation of unstable supracondylar humerus fractures in children. Technol Health Care.

[31] Horst M, Altermatt S, Weber DM, Weil R, Ramseier LE (2011). Pitfalls of lateral external fixation for supracondylar humeral fractures in children. Eur J Trauma Emerg Surg.

[32] Hohloch L, Konstantinidis L, Wagner FC, Strohm PC, Südkamp NP, Reising K (2016). Biomechanical comparison of different external fixator configurations for stabilization of supracondylar humerus fractures in children. Clin Biomechanics.

[33] Kwok SM, Clayworth C, Nara N (2021). Lateral versus cross pinning in paediatric supracondylar humerus fractures: a meta-analysis of randomized control trials. ANZ J Surg.

