# Supplementary figures and images for: Lateral entry pins and Slongo’s external fixation: which method is more ideal for older children with supracondylar humeral fractures?
Source: J Orthop Surg Res. 2021 Jun 21;16:396. doi: 10.1186/s13018-021-02541-z (PMC8215768; doi:10.1186/s13018-021-02541-z)

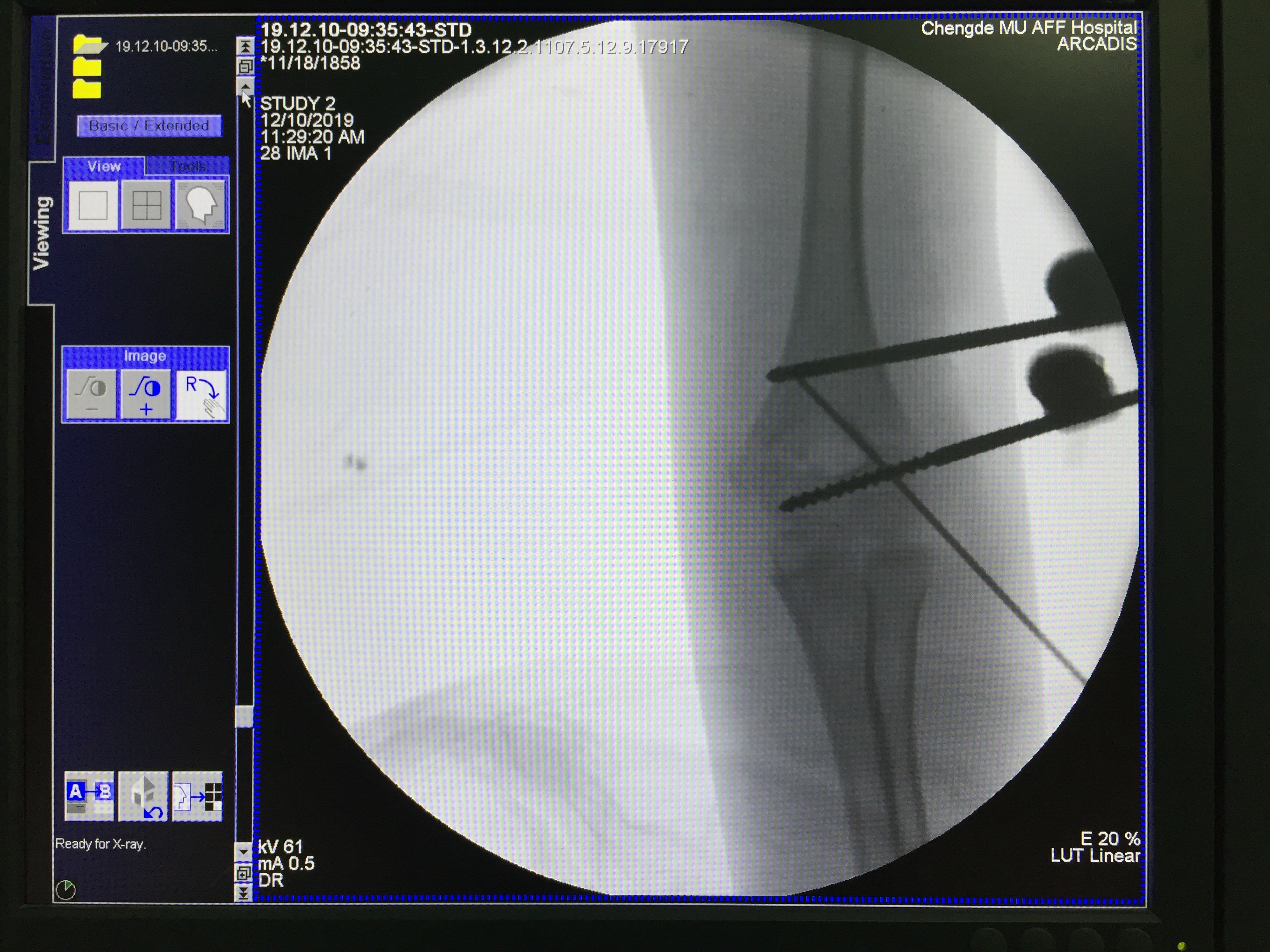

Supplement: Supplementary file 1 — Additional file 1. During operation. [file 13018_2021_2541_MOESM1_ESM.jpg]

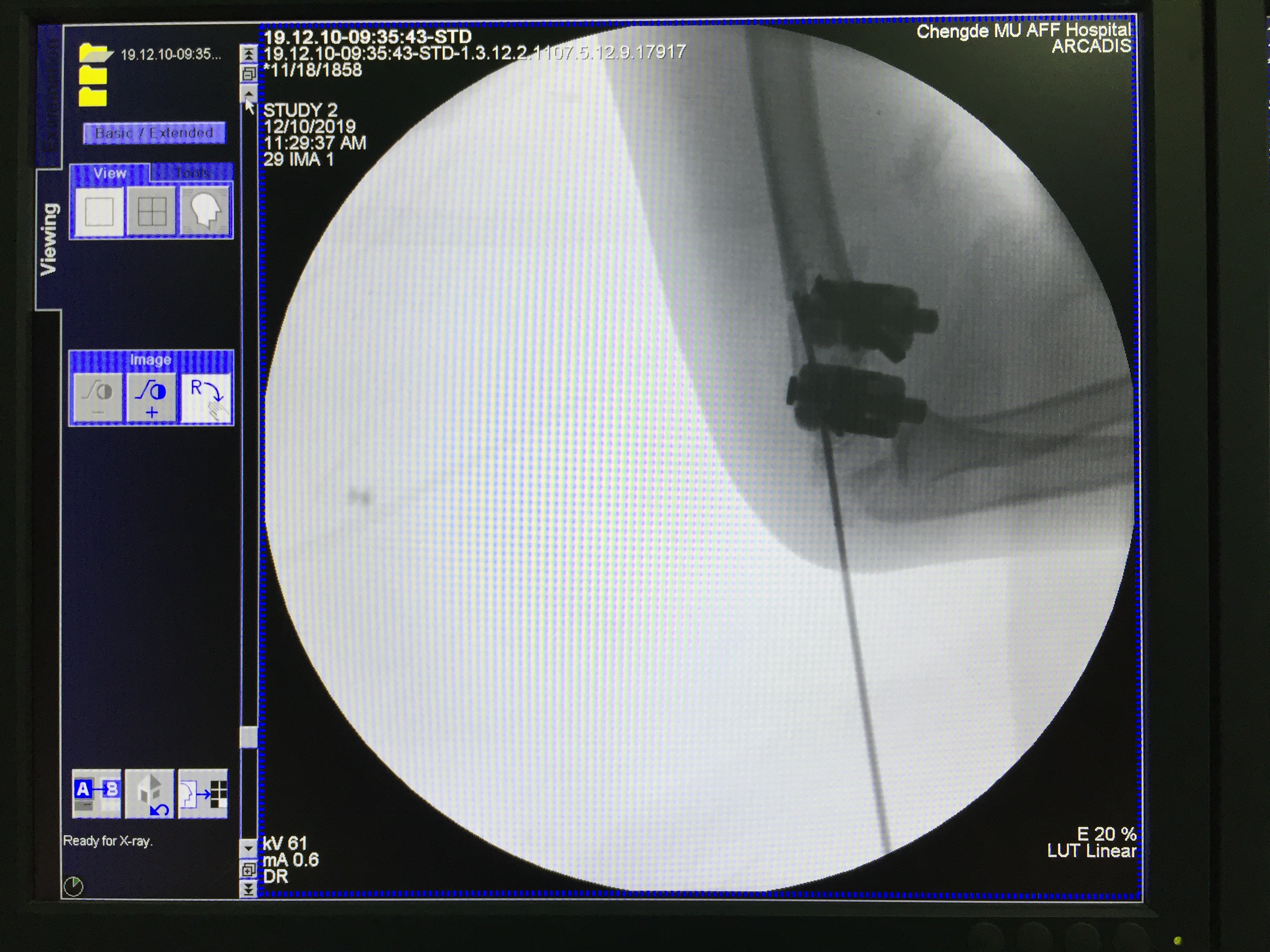

Supplement: Supplementary file 2 — Additional file 2. During operation. [file 13018_2021_2541_MOESM2_ESM.jpg]

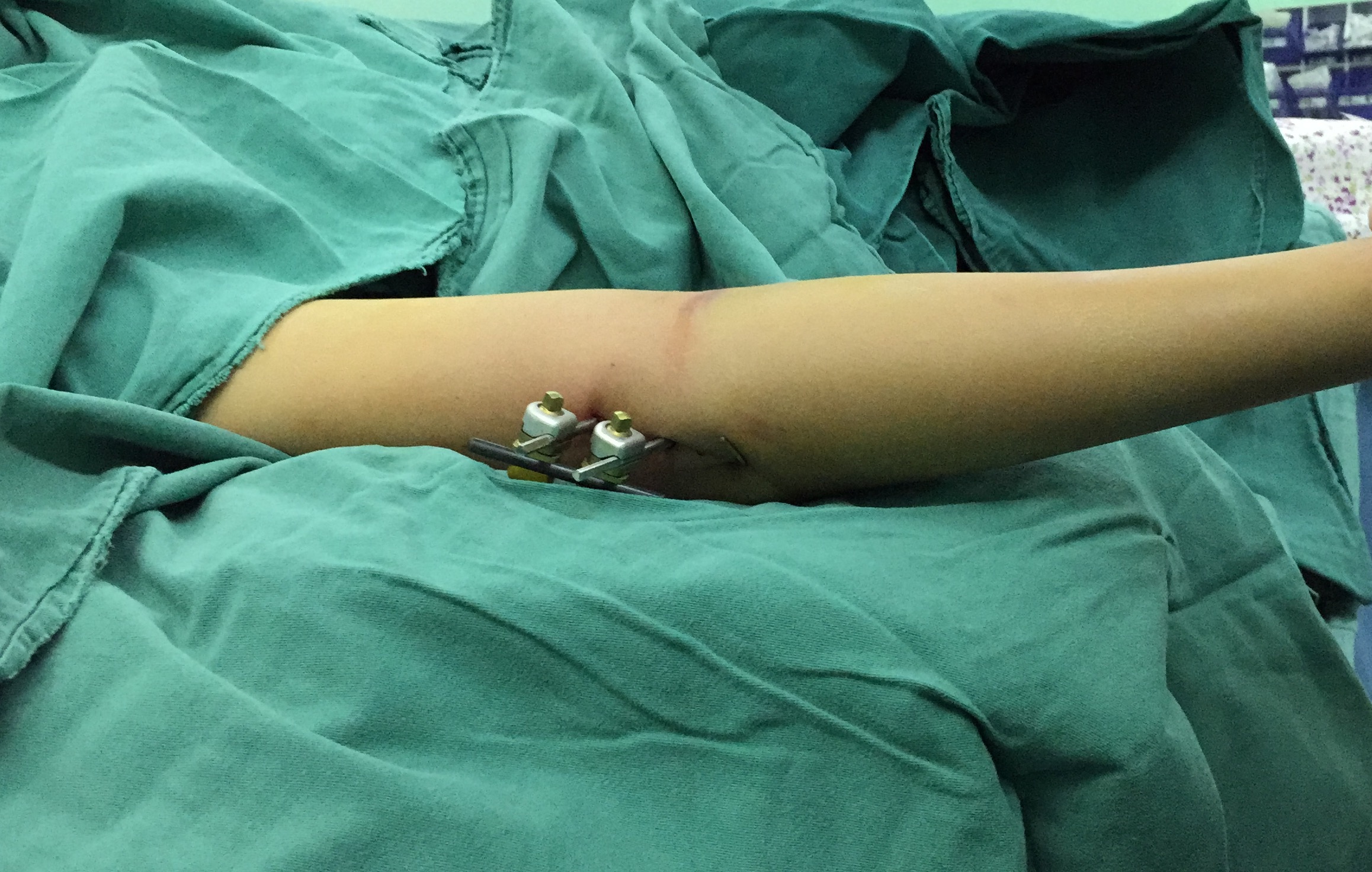

Supplement: Supplementary file 3 — Additional file 3. During operation. [file 13018_2021_2541_MOESM3_ESM.jpg]

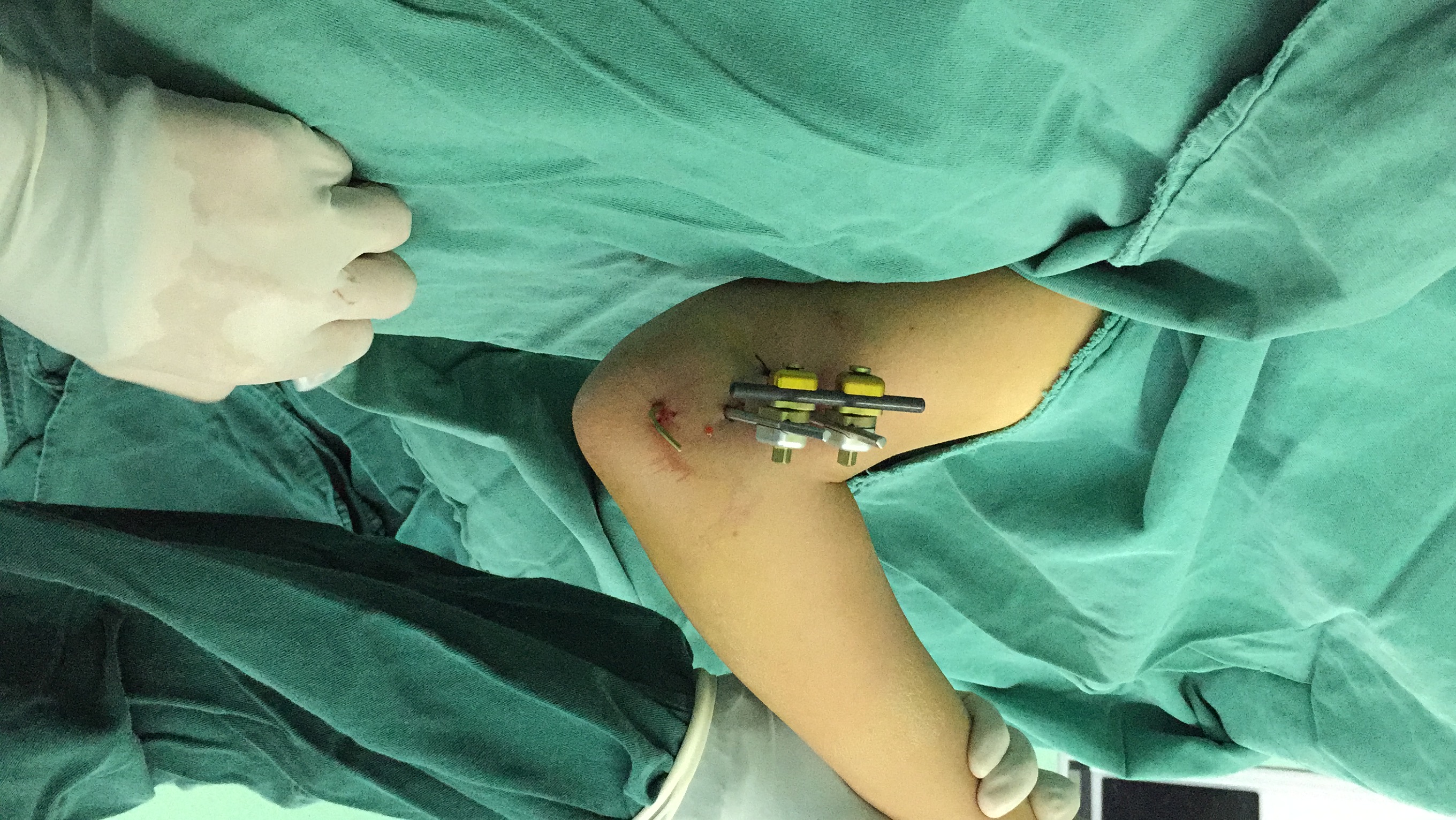

Supplement: Supplementary file 4 — Additional file 4. During operation. [file 13018_2021_2541_MOESM4_ESM.jpg]

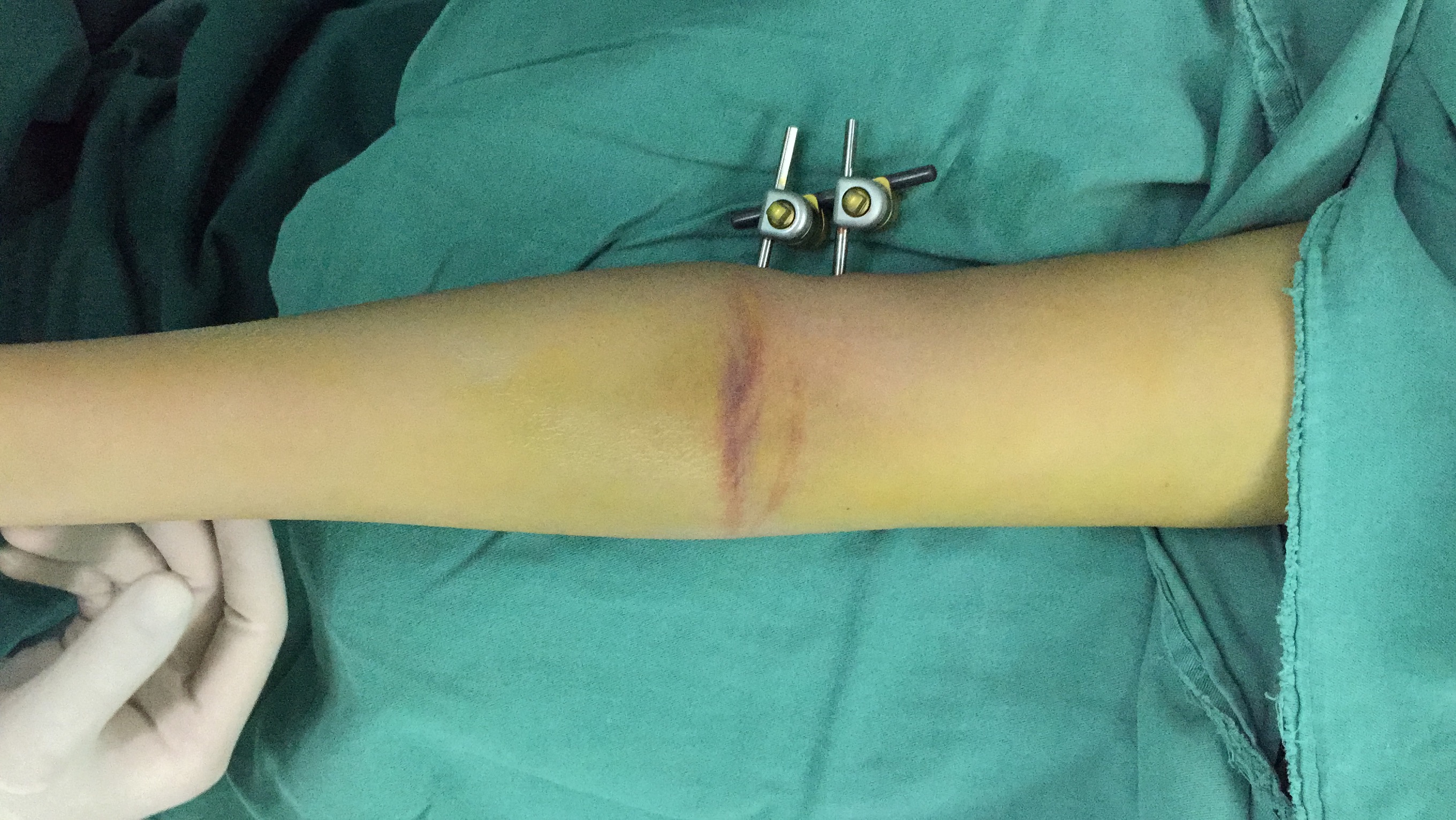

Supplement: Supplementary file 5 — Additional file 5. During operation. [file 13018_2021_2541_MOESM5_ESM.jpg]

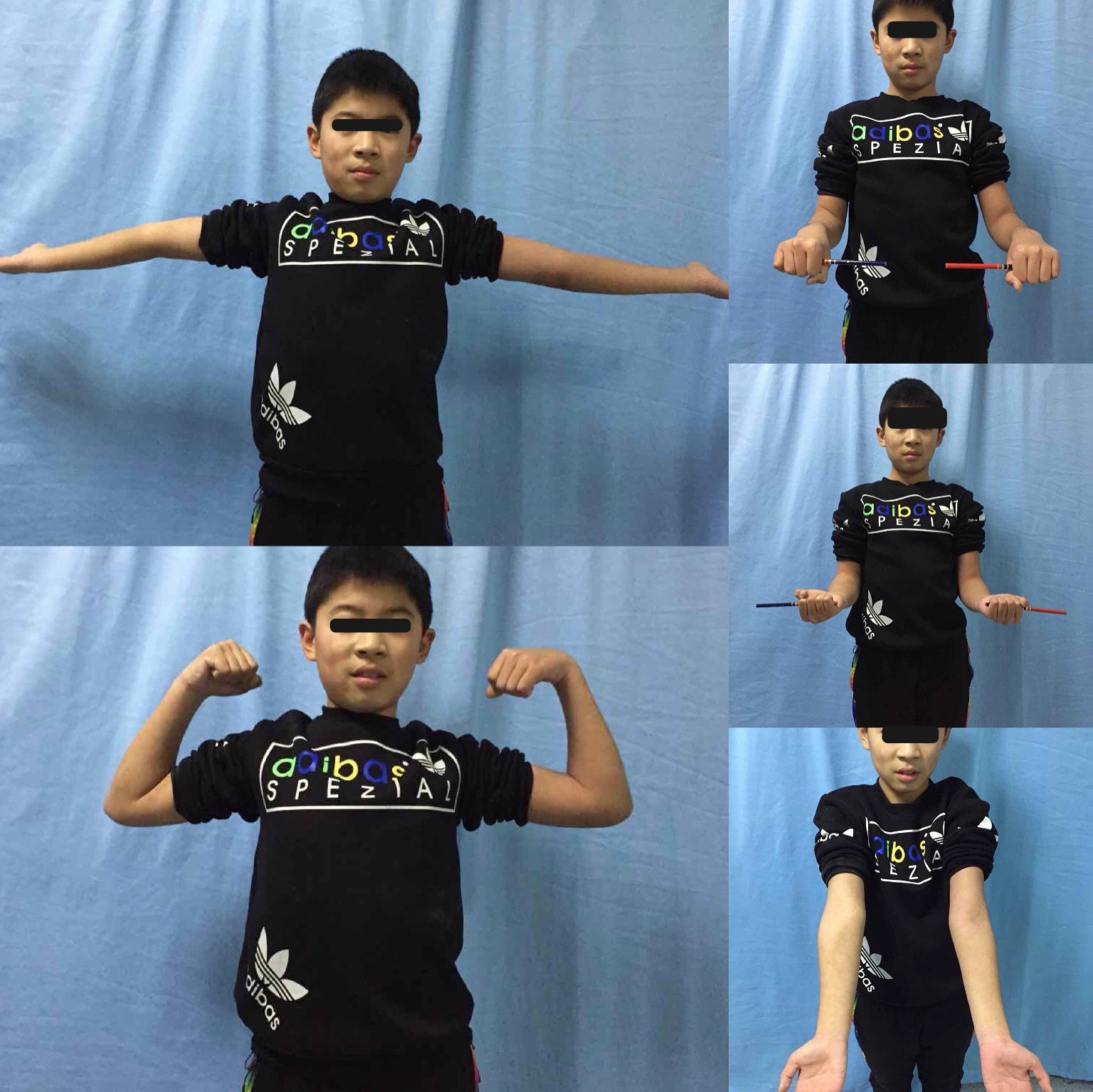

Supplement: Supplementary file 7 — Additional file 7. Post-operation. [file 13018_2021_2541_MOESM7_ESM.jpg]
